# Experimental Investigation on Thermal Conductivity and Thermal Diffusivity of Ex-Vivo Bovine Liver from Room Temperature down to −60 °C

**DOI:** 10.3390/ma14133750

**Published:** 2021-07-05

**Authors:** Elena Campagnoli, Valter Giaretto

**Affiliations:** Department of Energy, Politecnico di Torino, C.so Duca degli Abruzzi 24, 10129 Turin, Italy; valter.giaretto@polito.it

**Keywords:** bovine liver, thermal conductivity, thermal diffusivity, water content, density

## Abstract

Ex vivo animal tissues (e.g., bovine liver) as well as water-agar gel are commonly used to simulate both experimentally and numerically the response of human tissues to cryoablation treatments. Data on the low temperature thermal properties of bovine liver are difficult to find in the literature and very often are not provided for the whole temperature range of interest. This article presents the thermal conductivity and thermal diffusivity measurements performed on ex-vivo bovine liver samples using the transient plane source method. Regression coefficients are provided to determine these properties in different temperature ranges except for the phase transition during which no results were obtained, which suggests an ad hoc calorimetric analysis. A quick procedure is also suggested to determine the water mass fraction in the tissue. Moreover, an attempt to estimate the liver density in the frozen state using measurements performed solely at room temperature is also presented. The measured thermal conductivity and thermal diffusivity values are compared with data reported in literature highlighting a spread up to 40%. Moreover, it emerges that water-agar gel usually made with 2% by weight of agar does not show the same thermal properties as the bovine liver.

## 1. Introduction

Although the use of cryosurgery has gained importance in the past for the treatment of malignant tumors, [1,2,3,4] for example, and more recently for treating atrial fibrillation by catheter ablation, [5,6,7,8] for example, some controversial aspects about the cooling/heating dynamics are not yet fully understood. This matter is of primary importance and the clinical procedure to avoid recurrences and to prevent serious damages to the surrounding tissues, remains suboptimal.

In order to deepen these aspects, with reference to biological tissues and materials that mimic the thermal behavior of human tissues, several experimental investigations and numerical analyses were proposed over the years.

From a numerical point of view, the most used approach is based on the so-called bioheat equation and on the prediction of ice formation around the cryoprobe and its growth inside the tissue. In some cases both the metabolic heat and blood perfusion are neglected [9,10] and considered only for unfrozen region, because blood perfusion is blocked in frozen tissue and metabolic heat decreases exponentially [11]. The bioheat equation is also used to analyze the effects of multiple freeze-thaw cycles [12], showing that longer thawing periods are more effective due to recrystallization and osmotic damage produced.

The importance of the freeze-thaw sequence was also demonstrated through in-vivo experiments on porcine lung [13], which showed a significant change in the thermal properties of the tissue due to the bleeding caused by the thawing process, which in the subsequent freezing increases the size of the frozen region.

The in-vitro experiments on materials that mimic biological tissues are instead generally dedicated to the evaluation of the isothermal surface inside the ice ball surrounding the cryoprobe [14].

While most cryoablation models assume thermal effects and ice formation as a priority for the cell injury, a different approach is adopted in [15]. In this study the authors performed a sensitivity analysis on the thermo-physiological properties and on the parameters included in the bioheat equation, showing that if the cooling rate is not too high, the blood perfusion rate is the parameter that most influences the volume of the lesion during cryoablation.

Conversely, in clinical applications of cryosurgery for the treatment of atrial fibrillation by catheter ablation, the cooling rate is typically high, close to 120 K/min [6], and the optimal ablation temperature required for long-term success is lower than −50 °C [6,16]. In these contexts, the knowledge of the heat diffusion properties of the frozen tissue becomes important to evaluate the thermal field inside it and to properly set the duration of the procedure to avoid collateral damage.

The numerical analysis of heat transfer in tissues requires both to know some thermophysical properties and to know how these depend on the temperature.

The thermophysical properties of biological tissues are not easily found in the most recent literature, and the values reported are typically referred to temperature close to or above the ambient one [17]. Data relating to the properties of biological tissues below room temperature and when they are frozen, if available, are usually provided through correlations valid for narrow temperature ranges [18].

In the most recent literature, the values of the thermal properties of several biomaterials at cryogenic temperatures were proposed in [19], and as regards the thermal conductivity of water, water-agar gel and mouse liver were determined experimentally using the 3ω-method in [20].

In the numerical simulations, if the data relating to the thermal properties of the investigated tissue are not known, it is assumed that the values of the properties and their trend in temperature are the same as those shown by other biological tissues with an equivalent water content [21].

It is commonly believed that water-agar gel is able to mimic the thermal behavior of ex-vivo biological soft tissues and is therefore widely used for in-vitro investigations [22,23]. Since in a previous work the authors presented the temperature dependence of the properties for water-agar gel (2% agar) [24], it is essential to understand whether this material really mimics the thermal behavior of an ex-vivo biological tissue in the temperature range of interest.

With this aim, it was decided to investigate the ex-vivo bovine liver, assuming that it is the tissue whose morphology and internal structure are best reproduced by the water-agar gel.

As previously done for the water-agar gel, the experiments were performed from room temperature down to −60 °C, using the transient plane source method to measure the thermal conductivity and thermal diffusivity as a function of the temperature, and to consequently calculate the volumetric heat capacity.

In addition to compare the experimental results obtained for the ex-vivo bovine liver and the water-agar gel, the paper aims to define a suitable experimental procedure to investigate the thermophysical properties of a massive biological material, including water content and density, over a wide temperature range.

## 2. Materials and Methods

The measurements of the thermal properties of the ex-vivo bovine liver investigated were performed using a Hot Disk^®^ apparatus, model TPS 500 (Hot disk AB, Göteborg, Sweden). The device works using the transient plane source method, which allows measurement of thermal conductivity, thermal diffusivity, and consequently the calculation of the volumetric heat capacity of solid materials [24,25,26].

To perform the measurements at different temperatures, the experimental assembly was placed inside a climatic test chamber (CTC) that allows carrying out tests from −70 °C to +180 °C, with an accuracy in defining the set point of ±1 °C and a set point stability of ±0.1 °C.

With regards to the preparation of the sample and the experimental assembly for the measurements of the thermal properties, the methods suggested for determining the water content and density versus temperature are described in the following three paragraphs.

### 2.1. Preparation of the Sample and Experimental Assembly

The method used to perform the experiments and the procedure followed to prepare the experimental assembly is the same as described in [24] and was previously used to carry out measurements on water-agar gel samples.

The liver was handled to obtain an approximately cylindrical shape that almost completely filled the sample holder. Using a surgical scalpel, a cut was made in the central portion of the sample capable of accommodating the measurement sensor and the reference thermocouple. Two additional K-type sheathed thermocouples (outer diameter 1 mm), located at the bottom and top of the assembly, were used to control the temperature of the sample at its edges during the experiments.

**Figure 1:**
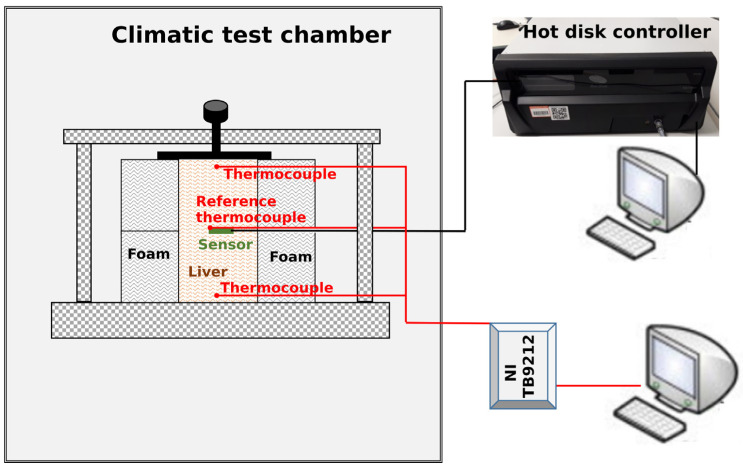
Schematic of the experimental set up showing a cross section of the experimental assembly with the liver placed in the sample holder made of polyurethane foam. The sensor and the three thermocouples used to monitor the experiment are also displayed.

**Figure 2:**
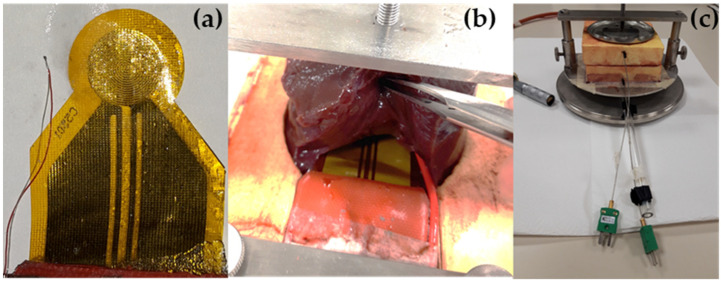
Experimental assembly: (**a**) sensor and reference thermocouple, (**b**) sensor and thermocouple positioned inside the liver sample, (**c**) experimental assembly ready for the measurement.

Figure 1 shows the schematic of the whole experiment, and Figure 2 displays the arrangement of the sensor, the three thermocouples, and the experimental assembly ready for the measurement.

### 2.2. Determination of the Water Content

The water content of the massive biological tissue was determined by measuring the mass loss by evaporation, obtained by keeping the sample at a fairly constant temperature around 80 °C and subjecting it to free convection at ambient conditions for an appropriate time interval. Compared to the initial mass, assuming the mass loss of the sample due solely to the evaporation of water, the value of the water content is complementary to the mass measured when dry.

The dehydration process versus time is expected to be asymptotic, and from a methodological point of view, it is necessary to comprehend the influence of the initial mass of the sample and the minimum time required to best evaluate the asymptotic value corresponding to the dehydrated sample.

Three samples of different mass and shape were produced from the same liver by picking the material from different positions. A sample is a few grams, approximately cubic in shape, with a side of about 15 mm; the other two are slices about 2 mm thick, with a mass of few tens of milligrams.

Using an analytical balance (Sartorius model Secura^®^ 125, self-calibrating with repeatability of ±0.01 mg, Sartorius, Goettingen, Germany), the initial mass $M_{0}$ was determined for each sample. Each of these was then placed on a heating plate, adjusted at the previous mentioned temperature, and reweighted at different time intervals, depending on the initial mass.

The dehydrated mass $M_{D}$ was considered achieved when the water content in the sample reached the equilibrium with that of the surrounding airflow. This condition was obtained for each sample after a proper time interval, when fluctuations not exceeding ±0.1 mg were observed around a value assumed as the dehydrated mass $M_{D}$.

With respect to the initial mass of the sample, the dehydrated mass fraction was found practically independent of the initial value, and in the range of 0.346–0.348. Therefore, an average water content of 0.652 ± 0.002 was assigned to this liver.

Taking into account that the time interval required to reach the asymptotic dry mass value is about a couple of hours for the small samples and ten times higher for the sample with an initial mass of few grams, a proper analysis of the mass loss transient can reduce the time required to a few tens of minutes.

**Figure 3:**
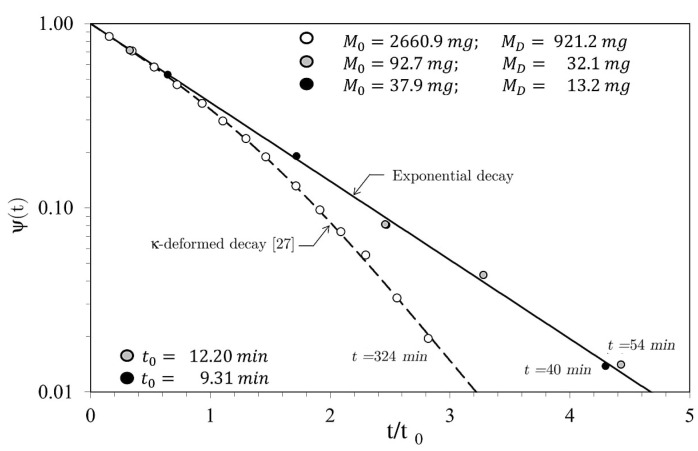
Dimensionless mass loss vs. dimensionless time for the lighter samples (continuous straight line) and for the heavier sample (dashed line).

Introducing the dimensionless mass loss ψ(t) as:

(1) $$\psi \left(t\right)=\frac{M\left(t\right)-M_{D}}{M_{0}-M_{D}},$$

where M(t) is the mass of the sample measured at time t, the plot in Figure 3 shows a different behavior between massive and non-massive samples as a function of time. Compared to massive cubic-shaped sample, the smallest, with a reduced aspect ratio (ratio between volume and effective evaporation surface), follows quite well an exponential decay with the following form:

(2) $$\psi \left(t\right)=\exp(-t/t_{0})\Rightarrow \ln\;\psi \left(t\right)=-t/t_{0}.$$

In Equation (2), $t_{0}$ is the characteristic time, and $-1/t_{0}$ is the slope in a semi-log diagram. Conversely, the loss of mass versus time of the heavier sample underlines a non-linear kinetics, in agreement with the k-deformed statistic introduced in [27].

The semi-log plot of the dimensionless mass variation ψ(t) versus the dimensionless time $t/t_{0}$ shown in Figure 3 refers to a large initial part of the transient and clearly displays the different behavior of the tested samples.

The solid straight line in the plot refers to Equation (2) and agrees with the dimensionless mass loss of the lighter samples. The diagram reports both the initial mass measured $M_{0}$ and the dehydrated mass $M_{D}$ for all the samples, while the characteristic time $t_{0}$ is shown for lighter samples only.

Although the analysis of the non-linear kinetics underlying the behavior of the massive liver sample is beyond the aim of this paper, the dashed line identifies the trend obtained by applying the theory detailed in [27].

In summary, to reduce the time needed to determine the mass loss by evaporation it is necessary to choose a low aspect ratio for the samples in order to obtain an exponential decay.

In this case, in a semi-log diagram, a linear trend is observed for the measured mass loss versus time. Consequently, both the asymptotic dehydrated mass $M_{D}$ and the characteristic time $t_{0}$ can be estimated by regression.

The mass M(t) in Equation (3), obtained by equating Equations (1) and (2), is a function of the dehydrated mass $M_{D}$ and the characteristics time $t_{0}$, which are the parameters to be estimated.

(3) $$M\left(t\right)=M_{D}\left[1-\exp(-t/t_{0})\right]+M_{0}\exp(-t/t_{0}).$$

### 2.3. Estimated Trend of Density Versus Temperature

Several essential hypotheses are required to determine density as a function of biological tissue temperature. Compared to the density value measured at room temperature, during cooling, the change in density is due only to the change in volume of the sample, since there is no mass variation.

Therefore, the dehydrated mass fraction $x_{D}=M_{D}/M_{0}$, and the complementary water fraction $x_{W}=1-x_{D}$ are constant during the process. Since the density variation is due only to the change in volume, the crucial hypothesis was to assign this effect only to water, assuming the density of the dehydrated tissue insensitive to the temperature variation in the investigated range.

The tissue volume reduction observed at the end of the dehydration process was evaluated to be approximately similar to that initially occupied by water at room conditions. For this reason, it was assumed that the changes in the tissue volume versus temperature were equal to the sum of the volume of the dehydrated tissue and the volume occupied by the water.

The volume of the dehydrated liver was supposedly temperature independent whereas the volume of the water was considered to be temperature dependent. In this way, by introducing the density of the dehydrated tissue $\rho_{D}$ and of water $\rho_{W}\left(T\right)$, the density of the tissue with respect to the temperature can be written as:

(4) $$\rho \left(T\right)={\left[\frac{x_{D}}{\rho_{D}}+\frac{x_{W}}{\rho_{W}\left(T\right)}\right]}^{-1}.$$

The density of frozen water is reported in the literature by Fukusako [28] and can be determined from the triple point down to −140 °C with the following relation where *T_0_ =* 273.15 K:

(5) $$\rho_{W}\left(T\right)=916.71-0.1073\;\cdot \;\left(T-T_{0}\right).$$

Above the triple point and up to 40 °C, the density of the water was determined using the following quadratic relation (*T*_0_ = 273.15 K):

(6) $$\rho_{W}\left(T\right)=999.8+0.0293\;\cdot \;\left(T-T_{0}\right)-0.0056\;\cdot \;{\left(T-T_{0}\right)}^{2}.$$

## 3. Results and Discussion

### 3.1. Evaluation of the Water Content

Five samples were used to determine water content in the liver. Samples 1.a, 1.b and 1.c are those used to evaluate the influence of the initial mass on the achievement of the dehydrated state, while samples 2 and 3 come from the liver of two other different animals. Table 1 reports for the dehydrated mass and the water fraction the values obtained experimentally with an estimated accuracy on the water mass fractions of about 1%.

Looking at Table 1, it is possible to see that the samples 1.a, 1.b, and 1.c, obtained from the same liver, have the same water content (water fraction $x_{w}=0.653$), while the fraction of water contained in the samples 2 and 3 is respectively about 4% and 9% higher. The maximum deviation from the average value of the water mass fraction (0.681) is 4.5%.

This fact seems to highlight a lack of homogeneity in water content between livers from different animals, which could, however, be simply linked to dehydration that occurred at a time prior to purchase.

### 3.2. Tests Performed Using the Hot Disk^®^ Apparatus

The tests were performed on three bovine liver samples from different animals, starting from room temperature down to −60 °C. The measurements were carried out during both the cooling of the sample and the subsequent phase of heating up to room temperature.

Since the authors are interested in evaluating the properties of the liver in the frozen state, the measurements were carried out starting from about 15 °C. The reason for the choice of this temperature is in the first place to allow, even at temperatures above freezing, the comparison of the properties of the liver with those of the water-agar gel previously investigated. A second reason is linked to the fact that the CTC does not regulate well around the ambient temperature and therefore does not allow to obtain a stable value to which to refers the measurement.

For each sample and each set point temperature, measurements were carried out by modifying the input thermal power supplied to the sensor or alternatively the duration of the pulse. The results reported here for thermal conductivity and diffusivity and for volumetric heat capacity are the average values obtained.

The graphs of these properties do not show values in the range −2 °C ÷ −13.9 °C as the measured data are not reliable. The indication of the fact that the measured properties are not acceptable is provided more than evidently by the volumetric heat capacity whose values are up to five times higher than those reported for water in the same temperature range.

This is for sure due to the supercooling of the water [29,30] contained in the liver. In fact, for the three tested samples, the phase transition begins at −2 °C, but does not end at the same temperature and develops down to about −14 °C.

This is certainly a limitation of the method used, which is not easily attributable to a single phenomenon. One possible explanation is that the heat released due to the phase change overlapped that generated by the Joule effect through the hot disk, disturbing the measurement signal and preventing any determination of the properties.

A second possible explanation can be related to the measurement method, which requires the thermal wave produced by the sensor to propagate in a single material medium. This condition is probably not met for one or both of the following reasons. First, during the phase transition, the thermal wave passes through parts of the material that have different levels of phase transition completion and consequently behave as if they were different materials. Secondly, during the transition, the electrical power supplied to the sensor, albeit small, produces a thermal power that is probably sufficient to thaw the tissue near the sensor, invalidating the measurement.

The analysis of the causes of this behavior is beyond the objectives of this research, but suggests performing an ad hoc calorimetric analysis to understand how the phase transition takes place and how the release of latent heat into temperature occurs.

**Figure 4:**
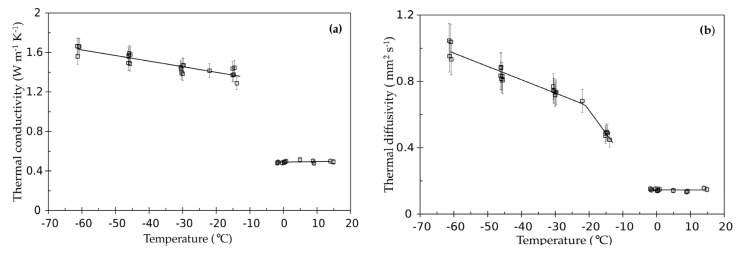
(**a**) Thermal conductivity and (**b**) thermal diffusivity of liver vs. temperature. The open squares represent the measured data while the solid lines are the trend lines obtained by performing linear regressions.

Figure 4a,b reports the thermal conductivity and thermal diffusivity data for the liver and the trend lines whose coefficients are shown in Table 2.

The error bars for the accuracy in the measurements of thermal conductivity are set, as declared for the TPS 500, equal to ±5%, while for the thermal diffusivity an error bar of ±10% is used based on the declared reproducibility.

The trend lines of the two properties as a function of temperature are practically flat at temperatures above 0 °C. On the contrary, for the frozen state the values of both the properties show an increasing trend as the temperature decreases. The trend line for the thermal diffusivity shows two different slopes above and below −22 °C. The different slope is probably not ascribable either to the thermal conductivity or to the density, which show a different but constant slope in the range −13.9 °C ÷ −60 °C.

Therefore, we should conclude that this behavior for the thermal diffusivity must be related to the specific heat capacity and consequently suggests that the phase transition is to be considered completed only around −22 °C.

Taking into account the accuracy associated to the measured properties, no effect can be imputed to the different water content. Furthermore, in the performed measurements, no evidence of an impact on the thermal properties due to the freezing and thawing cycles was detected.

**Figure 5:**
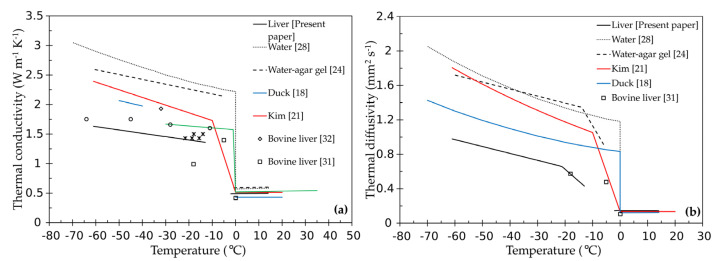
(**a**) Thermal conductivity and (**b**) thermal diffusivity trend lines for the measured liver samples compared with the literature data for water, water-agar gel, and ex-vivo liver of different animals.

Figure 5a,b shows a comparison between the trend lines obtained for the investigated liver, the trend lines for water [28], water-agar gel [24], and those obtained using correlations available in literature [18,21,31], as well as with data for ex-vivo liver of different animals [20,31,32,33].

Duck [18] reports for ex-vivo tissues two general correlations for calculating the thermal conductivity, the first in the range 0 °C ÷ 60 °C and the second in the range −40 °C ÷ −50 °C, as well as a correlation for thermal diffusivity (blue lines in the figures). In the correlation for the thermal conductivity in the unfrozen state, this property depends only on the water content, while in the frozen state it also depends on the temperature.

The equation for the thermal diffusivity given in [18] is a function of both the mass fraction of water inside the tissue and the value that the thermal diffusivity of the water assumes as the temperature varies. For these calculations, the water content was set equal to 68.1%, which is the average value that the authors measured in this work for the bovine liver.

Kim [21] used muscles properties data, considered equivalent to the liver in terms of water content, to simulate numerically (FEM) the cryoablation process and to compare the results with the experiments performed on ex-vivo bovine liver (red lines in the figures).

In this article [21], the thermal conductivity and thermal diffusivity are considered constant from room temperature down to 0 °C. On the contrary, for temperatures below −10 °C, the thermal properties are supposed to vary linearly with the temperature.

Above 0 °C, the results for the thermal diffusivity (Figure 5b) reported here, those mentioned in the literature for the ex-vivo tissue [18,21,32], and for both water [28] and water-agar gel [24], show a very good agreement. On the contrary, in the same temperature range, the results for the thermal conductivity are in good agreement only with the data reported by Kim [21] and Choi [31], while a deviation of about 12% with respect to the data reported by Duck [18] and by Valvano [32] is observed. Furthermore, a clear deviation can be observed between the thermal conductivity data for the ex-vivo liver and those for the water-agar gel that can be justified considering a difference in water content around 30%.

Thermal conductivity data, available in the literature for liver of different animals in the frozen state, show a remarkable spread. The data measured in the present study are about 40% lower than that reported by Kim [21] and about 15% lower than that available in the work by Choi [31], while they are in quite good agreement with the data by Lubner [20]. A higher deviation than that observed in the unfrozen state can be seen between the thermal conductivity of the ex-vivo liver and both water and water-agar gel.

In the frozen state, a similar disagreement was also observed for the thermal diffusivity values.

As mentioned above, no properties data were identified by the authors during the phase transition, i.e., in the range −2 °C÷ −13.9 °C, while data are reported in [21,31,32] over the same temperature interval. Beyond the thermal conductivity value reported in [32] at −5 °C, for sure related to the different experimental method, what is relevant are the different temperatures at which the phase transitions occur. In fact, in the case of liver, a behavior similar to that of water is proposed in [31], while in [21] the phase transition starts at 0 °C and ends at −10 °C.

### 3.3. Liver Density Dependence on Temperature

Density measurements were performed only at room temperature on three samples obtained from the same three bovine livers used in the previous experiments. Using Equation (4), the density $\rho_{D}$ of the dehydrated liver is determined as:

(7) $$\rho_{D}=\frac{1-x_{W}}{\frac{1}{\rho \left(T\right)}-\frac{x_{W}}{\rho_{W}\left(T\right)}},$$

where ρ(T) is the density of the liver at the temperature at which the measurement was performed, $\rho_{W}\left(T\right)$ is the water density calculated using Equation (6) at the same temperature, and $x_{W}$ is the water mass fraction reported in Table 1.

Using the density kit supplied with the Sartorius^®^ balance, which exploits the Archimedes principle, the density ρ(T) was determined weighting the samples both in air and immersed in distilled water and applying the following equation:

(8) $$\rho \left(T\right)=\frac{W_{a}\cdot \left[\rho_{w}\left(T\right)-\rho_{a}\left(T\right)\right]}{W_{a}-W_{w}}+\rho_{a}\left(T\right),$$

$W_{a}$ and $W_{w}$ are the weights in air and water, respectively, and $\rho_{a}\left(T\right)$ is the density of air evaluated using the ideal gas state equation. Temperature *T* is measured inside the distilled water using a bulb thermometer. The results are shown in Table 3.

The average value for the density of the dehydrated liver was determined using the values of only samples 2 and 3, since a more relevant release of serum was observed when sample 1 was immersed into the water. Therefore, the average value for the density of the dehydrated liver used in the following analyses is $\rho_{D}=1430\pm 22\;kg\;m^{-3}$.

**Figure 6:**
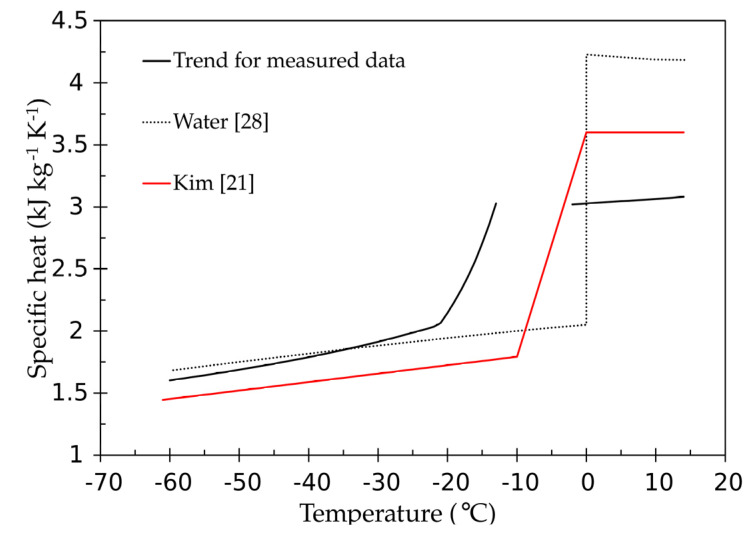
Comparison between the temperature dependence of the specific heat of the liver obtained in the present work and the literature data for the liver and pure water.

Using Equation (4) to determine the liver density versus temperature, and the trend line for the volumetric heat capacity displayed in Table 2, an attempt to evaluate the temperature dependence of the specific heat is plotted in Figure 6. In this figure, the data for pure water and those proposed by Kim [21] are compared with the specific heat obtained here for the ex-vivo bovine liver.

Quite similar behavior is observed for the frozen liver and water below −20 °C, whereas a remarkable difference is observed for the unfrozen state, well above 45%. An overall disagreement is also observed about the specific heat data reported by Kim [21].

A limitation in this survey derives directly from the experimental setting. In fact, the use of an ex vivo tissue does not allow taking into account effects linked to blood perfusion and metabolic heat.

## 4. Conclusions

In summary, this study reports the thermal conductivity and thermal diffusivity of the ex-vivo bovine liver from room temperature down to −60 °C, except for the temperature range during which the phase transition occurs. The coefficients used to draw the linear trend lines of these two properties are also provided, allowing the calculation of the volumetric heat capacity.

The measured values are compared with data available in the literature and with both those for pure water and water-agar gel. A remarkable spread up to 40% can be noticed with respect to the literature data. Furthermore, it emerges that the water-agar gel (2% agar) does not have the same thermal properties as the bovine liver neither in the unfrozen nor in the frozen state, suggesting the use of different percentages of agar powder to better mimic this biological material.

An attempt is also presented to determine the temperature dependence of the density of the liver simply based on: the water content in the liver and its density at room temperature as well as the temperature dependence of the water density.

A reliable estimate of liver-specific heat was not possible in the present work, but will be considered for future studies. Further investigations will also be devoted to the analysis of the phase transition to understand better how the latent heat of solidification is released.

## Figures and Tables

**Table 1:** Water fractions for the examined samples: initial mass $M_{0}$, dehydrated mass $M_{D}$, and water fraction $x_{w}$.

Sample	*M*_0_ (mg)	$M_{D}$ (mg)	$x_{w}$ (-)
1.a	2660.9	921.2	0.654
1.b	92.7	32.1	0.654
1.c	37.9	13.2	0.652
2	998.1	321.4	0.678
3	40.4	11.6	0.713

**Table 2:** Linear regression coefficients (*T_0_* = 273.15 K).

Thermal Properties	Coefficients
Conductivity λ(T), W/(m K) $\lambda \left(T\right)=\lambda_{0}\left[1+b_{\lambda }\left(T-T_{0}\right)\right]$	$-1.9\;{}^{\circ}C\leq \left(T-T_{0}\right)\leq 14\;{}^{\circ}C$ $\lambda_{0}=0.4900\;W/\left(mK\right)$ $b_{\lambda }=0.0010\;\left(K^{-1}\right)$ $-61\;{}^{\circ}C\leq \left(T-T_{0}\right)\leq -13.9\;{}^{\circ}C$ $\lambda_{0}=1.2861\;W\left(mK\right)$ $b_{\lambda }=-0.0044\;\left(K^{-1}\right)$
Diffusivity α(T), mm^2^/s $\alpha \left(T\right)=\alpha_{0}\left[1+b_{\alpha }\left(T-T_{0}\right)\right]$	$-1.9\;{}^{\circ}C\leq \left(T-T_{0}\right)\leq 14\;{}^{\circ}C$ $\alpha_{0}=0.1458\;mm^{2}/s$ $b_{\alpha }=-0.0002\;\left(K^{-1}\right)$ $-22\;{}^{\circ}C\leq \left(T-T_{0}\right)\leq -13.9\;{}^{\circ}C$ $\alpha_{0}=0.0696\;mm^{2}/s$ $b_{\alpha }=-0.3998\;\left(K^{-1}\right)$ $-61\;{}^{\circ}C\leq \left(T-T_{0}\right)\leq -22\;{}^{\circ}C$ $\alpha_{0}=0.4891\;mm^{2}/s$
Volumetric specific heat ρc(T), MJ/(m^3^ K)	$\rho c\left(T\right)=\frac{\lambda_{0}\left[1+b_{\lambda }\left(T-T_{0}\right)\right]}{\alpha_{0}\left[1+b_{\alpha }\left(T-T_{0}\right)\right]}$

**Table 3:** Density of fresh and dehydrated liver: $W_{a}$ weight in air, $W_{w}$ weight in water; density of the liver fresh ρ(T) and dehydrated $\rho_{D}$.

Sample	*T* (°C)	*W_a_* (mg)	*W_w_* (mg)	*ρ(T)* (kg m^−3^)	*ρ_D_* (kg m^−3^)
1	22.5	1425.8	1291.1	1112	1420
2	24	1846.9	1689.9	1101	1408
3	24	2021.1	1857.6	1096	1452

## Data Availability

Not applicable.

## Abbreviations

CTC	Climatic test chamber
c	Specific heat (kJ/kg K)
*M*	Sample mass (mg)
*M_D_*	Dehydrated sample mass (mg)
*M*_0_	Sample initial mass (mg)
*T*	Temperature (K)
*T*_0_	Reference temperature (K)
*t*	Time (s)
*t*_0_	Characteristic time (s)
*x_D_*	Dehydrated mass fraction (-)
*x_W_*	Water fraction (-)
**Greek symbols**	
Ψ	Dimensionless mass loss (-)
*ρ*	Density (kg/m^3^)
**Subscripts**	
*D*	Dehydrated
*W*	Water
